# Bioelectric State and Cell Cycle Control of Mammalian Neural Stem Cells

**DOI:** 10.1155/2012/816049

**Published:** 2012-09-11

**Authors:** Julieta Aprea, Federico Calegari

**Affiliations:** DFG-Research Center and Cluster of Excellence for Regenerative Therapies, Medical Faculty, Dresden University of Technology, Fetscherstrasse 105, 01307 Dresden, Germany

## Abstract

The concerted action of ion channels and pumps establishing a resting membrane potential has been most thoroughly studied in the context of excitable cells, most notably neurons, but emerging evidences indicate that they are also involved in controlling proliferation and differentiation of nonexcitable somatic stem cells. The importance of understanding stem cell contribution to tissue formation during embryonic development, adult homeostasis, and regeneration in disease has prompted many groups to study and manipulate the membrane potential of stem cells in a variety of systems. In this paper we aimed at summarizing the current knowledge on the role of ion channels and pumps in the context of mammalian corticogenesis with particular emphasis on their contribution to the switch of neural stem cells from proliferation to differentiation and generation of more committed progenitors and neurons, whose lineage during brain development has been recently elucidated.

## 1. Introduction

An extensive literature has described the features and properties of bioelectric gradients and signaling in a variety of tissues of many species during development, adulthood, and regeneration [1–5]. In particular for the developing nervous system, it has become clear that the concerted action of membrane channels and ion pumps establishing a resting membrane potential (*V*_mem_) and other bioelectric parameters plays important roles in migration, survival, maturation, and functionality of newborn neurons [6–8]. Certainly less investigated is whether similar parameters may also play a role in controlling the switch of neural stem and progenitor cells (altogether referred to as NSC) from proliferative to neurogenic divisions but various evidences have accumulated in recent years making this possibility likely; in particular, when considering the multiple factors coupling bioelectric gradients and cell cycle progression [1, 7, 9, 10] as well as the effects of cell cycle length on proliferation versus differentiation of neural, and other somatic, stem cells [11, 12].

**Figure 1:**
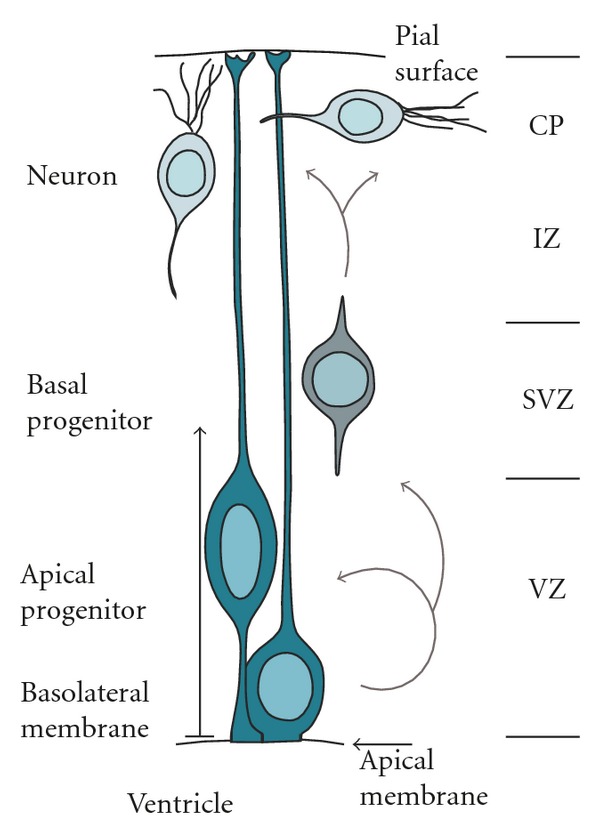
Scheme representing cell types in the developing mammalian cortex with (from top to bottom) neurons, basal (BP), and apical (AP) progenitors forming the cortical plate (CP), intermediate (IZ), subventricular (SVZ), and ventricular (VZ) zones, respectively. Lineages are depicted (arrows). Note the distinction between apical and basolateral plasma membrane of AP establishing the apicobasal polarity of the developing cortex.

However, the limits of our knowledge in this area are particularly evident during mammalian brain development in which the establishment of new, sophisticated tools has only recently allowed the characterization of the physiological lineage of NSC. Specifically, during embryonic development of the mammalian cortex, polarized radial-glial cells, also referred to as apical progenitors (AP) forming the ventricular zone (VZ), progressively switch from divisions that generate additional AP to divisions that generate more committed, neurogenic progenitors leaving the VZ to form the subventricular zone (SVZ) at its pial, or basal, boundary; hence the name basal progenitors (BP) [13, 14]. BP lose polarity, have limited self-renewal potential, and are soon consumed through symmetric neurogenic divisions to generate a pair of postmitotic neurons that migrate towards the pial surface to form the various neuronal layers of the mammalian cortex [13, 14] (Figure 1). Currently, most mammalian cortical neurons are thought to be derived from BP, rather than AP, and, interestingly, the appearance of this subpopulation of cells specifically in mammals has been proposed to be a critical step through which the massive enlargement in cortical surface area has been achieved during evolution of our species [15–17].

Unfortunately, major technical limitations in investigating the role of bioelectric signals in neurogenic commitment during development have prompted most groups to use nonmammalian organisms, lacking BP, as model systems. Moreover, of the few reports in which mammalian NSC have been used, the vast majority were carried out in cultures of dissociated cells in which the loss of positional information and polarity makes it difficult to identify and compare characteristics of AP and BP. For these reasons, our knowledge about bioelectric signaling during mammalian brain development is very limited and its role in controlling the switch from proliferating AP to neurogenic BP can only be retrospectively inferred from previous studies in which these questions were, if any, only indirectly addressed.

Other authors have already summarized our current knowledge about a potential role of bioelectric signaling in stem cell function in various tissues or, within the nervous system, without considering progenitor lineages of the mammalian cortex [1–4, 7–9]. Thus, in this paper we attempted to make the retrospective links that may help address its role in the switch of mammalian NSC from proliferation to neurogenesis, which is fundamental towards understanding brain development and, perhaps, designing novel approaches of therapy of the mammalian central nervous system. Considering the extensive breadth of this area of research, we decided to focus our attention exclusively on the role of ion channels and pumps and their role in establishing a resting membrane potential in mammalian NSC of the developing embryo without discussing other bioelectric features, such as capacitance and input resistance, or additional roles of ion channels and metabotropic transporters involved in intracellular Ca^2+^ signaling, that were discussed elsewhere [1–8].

## 2. Establishing a Resting Membrane Potential in NSC

The fundamental mechanisms controlling the resting membrane potential of NSC are essentially identical to those of any other cell type being regulated by the permeability of ion channels and the activity of ion pumps and exchangers establishing ion gradients across membranes [8, 18–20]. Members of the first group include “leak” as well as voltage- and ligand-gated channels that allow the passive diffusion of ions through membranes after opening as a result of a change in voltage or binding to a specific ligand, respectively [18, 20]. Examples of the second group include various types of ATPases and other enzymatic complexes exchanging ions through membranes against their gradients by consumption of energy, including the ubiquitous Na^+^/K^+^, H^+^, Ca^2+^ ATPases, and Na^+^/K^+^/Cl^−^ cotransporters covering almost the whole spectrum of biologically relevant ions [18, 19]. The roles of the three major classes of ion channels and pumps in embryonic mammalian neurogenesis will be discussed separately.

### 2.1. Voltage-Gated Ion Channels

The study of voltage-gated ion channels has been particularly important for understanding the origin of action potentials, but recent evidences suggest that they may play important roles also in nonexcitable cells such as NSC. Several channels responsible for establishing a *V*_mem_ in NSC during development [21] and adulthood [22] have been characterized, but many discrepancies and uncertainties remain with regard to the specific features of the bioelectric state and signaling in different subpopulations of NSC. In particular, of the main types of voltage-gated K^+^ currents both outward, delayed rectifier [21, 23–25] and inward rectifier currents in response to hyperpolarizing pulses [21] were detected. On the other hand, fast, A-type, transient outward K^+^ currents were detected in NSC cultures [21, 23–25] but not in the VZ of organotypic slices [26].

Since the resting membrane potential of most animal cells is slightly higher than the reversal potential of K^+^, the overall effect of blocking K^+^ channels is to promote depolarization; this most typically correlates with increased cell proliferation. In fact, voltage-gated K^+^ currents are involved in the regulation of the ce ll cycle, in particular G1, in many cell types [1, 7, 9, 10, 27], including NSC [8, 22], and treating NSC with certain K^+^ channels antagonists promoted their proliferation in a number of assays [23, 28–30]. For example, blockage of voltage-gated delayed rectifier K^+^ channels in cells isolated from rat midbrain increased the proportion of dividing precursors from ca. 10 to 30% [23]. In 12-week human fetal NSC, inhibition of delayed-rectifier K^+^ channels either did not affect proliferation or increased it, depending on the blocker used, while inhibition of A-type K^+^ channels impaired cell viability [29]. This proliferation effect of certain blockers of K^+^ channels has also been confirmed in adult neurogenic or embryonic gliogenic progenitors in which increased or decreased proliferation has been observed depending on which particular subtype of K^+^ channels has been blocked [28, 30]. Along these lines, a block in K^+^ channels in oligodendrocyte progenitors correlated with inhibited proliferation and an increase in the levels of the G1-specific cyclin-dependent kinase inhibitors p21 and p27 [31]. 

Reconciling these partly contrasting results is difficult not only because the pharmacological approaches and origin of NSC varied among studies but also because effects on membrane potential as a result of a block of one given type of K^+^ channel were rarely measured to corroborate effects on de- versus hyperpolarization.

The K^+^ currents found in NSC are also present in immature neurons in which inward Na^+^ currents soon appear that increase their amplitude during neuronal maturation until reaching values characteristic of mature neurons [26, 32]. In contrast to K^+^, the presence of voltage-gated Na^+^ channels in NSC is controversial. Several studies have detected low Na^+^ currents in NSC preparations, but these were present only in a relatively small subpopulation of cells of which most have been classified as early born neurons [21, 33], a conclusion that was also corroborated by direct measurement of NSC in the VZ of mouse cortical slices [34]. Nevertheless, it cannot be excluded that Na^+^ currents may appear in more committed neurogenic progenitors, such as BP, since these cells were reported to initiate the expression of genes characteristically identifying postmitotic neurons [35, 36]. This possibility is consistent with the detection of Na^+^ current in only a subpopulation of cortical progenitors [33] and is not invalidated by the absence of Na^+^ currents in the VZ [34] since this latter study was limited to cells with radial morphology, that is, AP. 

Direct evidence for the appearance of depolarizing Na^+^ currents in the transition from AP to BP can be retrospectively inferred from a study by Bahrey and Moody in which organotypic slice cultures from the embryonic day (E) 14 mouse brain were used to measure bioelectrical parameters of different progenitor subtypes [26]. Upon labeling with vital dyes, the authors could identify radial versus nonradial cells within the VZ observing a bias for the presence of Na^+^ currents in the latter population [26]. Interestingly, the proportion of cells in the VZ displaying Na^+^ currents increased during development from 0, 30, and then 50% at E9, E14 and E16, respectively [26]. Not only these values fit remarkably well with the proportion of BP detected in the VZ [37], but one year after the study by Bahrey and Moody three independent reports could also demonstrate that the vast majority of nonradial cell in the VZ are, indeed, BP [38–40]. Thus, it can now be retrospectively concluded that the work by Bahrey and Moody provided the first strong evidence for a difference in AP versus BP currents at a time when, remarkably, cellular and molecular features of BP were not even characterized.

Similar to K^+^, the presence of voltage-gated Ca^2+^ currents in NSC is consistently reported by various studies. Inward currents were detected in cells within the VZ in brain slices and dissociated cultures [26, 41, 42], and since similar currents were elicited also in preparations from E10 rat spinal cord [21], that are known to lack BP, it is likely that voltage-gated Ca^2+^ channels are a feature of all NSC. In cells isolated from human embryonic central nervous sytem [43] and P0 mouse cortex [44] small Ca^2+^ currents were only detected upon differentiation conditions in cells with neuronal morphology. Treating NSC with blockers of L-type voltage-gated Ca^2+^ channels has been found to reduce the number of neurons in differentiation conditions while, conversely, activating the channels triggered the opposite effect [44]. Yet, since the same experiments failed to detect Ca^2+^ currents in undifferentiated NSC [44], it may be concluded that the different number of neurons detected in this study may be attributed to effects on neuronal survival or an altered timing in the expression of neuronal markers in postmitotic cells rather than to a change in the fate of NSC proper.

The expression pattern during development of the fourth type of ion channels, Cl^−^, has also been described [45], but functional experiments on their role in NSC differentiation are missing.

**Figure 2:**
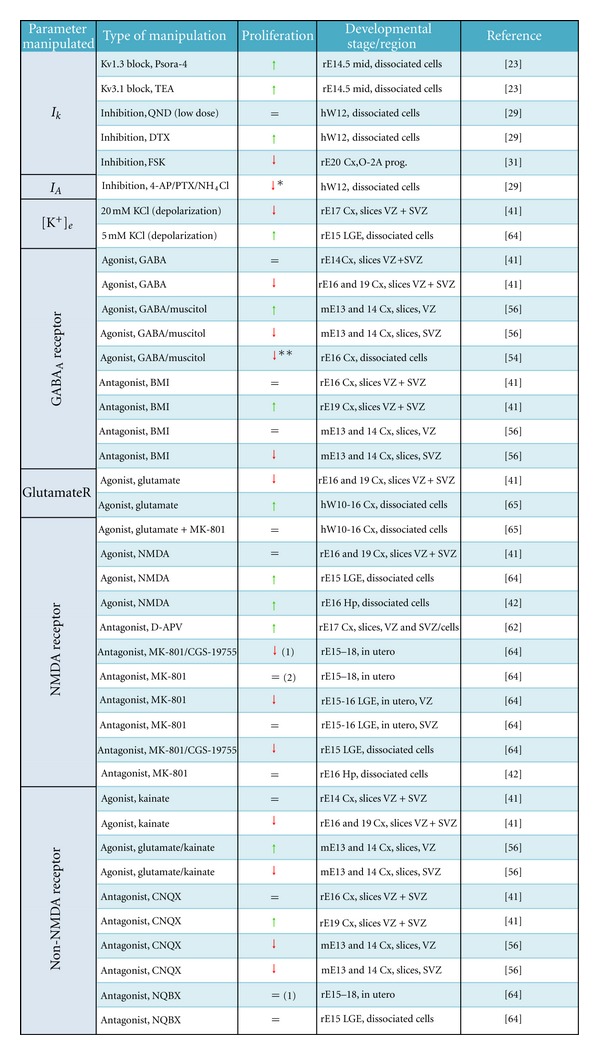
Effects upon manipulation of ion channels or extracellular ionic composition on the proliferation of NSC. Green and red arrows indicate increased or decreased proliferation, respectively, as deduced from incorporation of thymidine analogues or number of neurons in the adult striatum (1) or motor cortex (2). Agonists and antagonists used (Psora-4 = 5-(4-phenylbutoxy)psoralen; TEA = tetraethylammonium chloride; QND = quinidine; DTX = *α*-dendrotoxin; FSK = forskolin; 4-AP = 4-aminopyridine; PTX = phrixotoxin; BMI = biculline methionine; D-APV = D(-)-2-amino-5-phosphonopentanoicacid; CNQX = 6-cyano-7-dinitroquinoxaline-2,3-dione; NQBX = 1,2,3,4-tetrahydro-6-nitro-2,3-dioxo-benzol(f)-quinoxaline-7-sulfonamide) as well as source of NSC form different species (r = rat; h = human; m = mouse), developmental stage (E = embryonic day; W = embryonic week), or region (mid = midbrain; Cx = cortex; LGE = lateral ganglionic eminence; Hp = hippocampus; VZ = ventricular zone; SVZ = subventricular zone) are indicated. *reduced viability; **only in the presence of bFGF; O-2A = oligodendrocyte progenitors.

Altogether, several reports point to a role of voltage-gated ion channels in NSC proliferation with K^+^ channels being more consistently implicated in this process. Most studies in this area were performed using dissociated cells, or in slice cultures but without considering different progenitor subtypes, thus making it difficult to infer differences between AP and BP; the latter probably being characterized by the presence of Na^+^ currents [26]. It has been suggested that depolarization has a positive effect on proliferation [1, 7, 9], and many of the studies discussed above extend this view to NSC via manipulations that alter the activity of voltage-gated ion channels (Figure 2). Yet, the molecular mechanisms underlying this correlation are unknown. 

### 2.2. Ligand-Gated Ion Channels

The study of ligand-gated ion channels is most typically associated with the understanding of neurotransmitter-dependent excitability of neurons and neuroendocrine cells, but evidences collected over the years have shown that at least two ligands, *γ*-aminobutyric acid (GABA) and glutamate, play important roles in NSC activity not only in the adult [46] but also in the developing brain even before functional synapses are formed [41, 47, 48].

Among the most extensively studied, the GABA_A_ receptor is a ligand-gated Cl^−^ channel and because of the particular pattern of Cl^−^ transporters expressed in NSC of the embryonic brain [49, 50], these cells present a low Cl^−^ reversal potential implying that during development GABA depolarizes NSC and immature neurons instead of hyperpolarizing them as it does in the mature brain [48]. Functional GABA_A_ receptors and GABA are expressed in mammalian NSC during brain development [41, 51] acting through paracrine/autocrine signaling [47, 52, 53], and NSC in the VZ start to respond to GABA by depolarizing *V*_mem_ at E15 but not before [41]. The cellular origin of nonsynaptic GABA release is still controversial, but there is evidence for the presence of a synthetic machinery in NSC [51].

Several studies have pointed to an effect of GABA on NSC proliferation. LoTurco et al. found that GABA inhibits DNA synthesis in embryonic rat cortical explants and since GABA caused a reversible increase in [Ca^2+^]_i_, and depolarization by K^+^ had similar effects to GABA, the authors hypothesized that the effect on proliferation is mediated through activation of voltage-gated Ca^2+^ channels [41]. Consistently, GABA administration on dissociated cells was found to inhibit proliferation while promoting differentiation [54, 55]. On the other hand, Haydar et al. subsequently observed that GABA increased proliferation with a shortening of the cell cycle and decreased differentiation in the VZ while, interestingly, the opposite effect was found in the SVZ, where BP reside [56]. Clearly, differences between AP and BP were lost when NSC were pulled together [41] or studied using dissociated cultures [54, 55]. Other reports have also pointed to a positive effect of GABA on proliferation [57], and a vast literature has described its many effects on survival, migration, maturation, and synaptogenesis of newborn neurons [48, 52, 53].

Glutamate is the principal excitatory neurotransmitter in the adult cerebral cortex whose signals, as for GABA, are mediated by ionotropic and metabotropic receptors. The former group is further subdivided into three types based on their pharmacological and electrophysiological properties and named after their specific agonists: NMDA, AMPA, and KA receptors [58]. Considering the similar effect of GABA and glutamate on depolarization, it is not surprising that both neurotransmitters elicited similar effects on NSC.

The presence of NMDA receptors in NSC has been reported in several systems including NSC lines, primary cultures, and organotypic slice preparations displaying low expression levels of this receptor and small NMDA-mediated currents [59–62]. The fact that the NMDA-elicited currents observed in the VZ are all but a small fraction of those in the cortical plate, where neurons reside, [62] may explain why these currents were not detected by previous studies [41].

One early work reported increased proliferation upon block of NMDA receptors in NSC of the VZ and SVZ in slice cultures [62], but later studies consistently reached the opposite conclusion by showing that agonists of NMDA receptors increase proliferation while, conversely, antagonists trigger the opposite effect in vitro and in vivo [42, 63–65].

AMPA and KA receptors are expressed during development as early as E10 [60, 66]. In rat cortical slices, an increasing proportion of cells in the VZ depolarized upon AMPA or KA exposure as a function of developmental time from 0 to 100% between E14 and E16 [41]. Exposure to KA in rat cortical slices decreased NSC proliferation [41], while exposure to AMPA or KA agonists in mouse cortical slices shortened the cell cycle in the VZ but had the opposite effect on the SVZ while inhibiting neurogenesis [56].

Other ionotropic receptors for glycine, acetylcholine, and serotonin have also been implicated in neuronal development but primarily in the maturation, migration, synaptogenesis, and circuit formation of postmitotic neurons rather than in the regulation of NSC proliferation versus differentiation proper [52, 53].

Most of the limitations discussed in the context of voltage-dependent ion channels with regard to their involvement in AP to BP transition during mammalian cortical development hold true for ligand-dependent ion channels, and equally valid is the overall positive correlation between manipulations that depolarize NSC and increased proliferation (Figure 2).

### 2.3. Ion Pumps

The third big family of proteins essential for establishing an electric potential across membranes comprises enzymatic macrocomplexes converting energy, most typically chemical in the form of ATP or electrochemical gradients, to pump ions against their concentration gradient [18, 19]. An additional, and equally important, role of certain pumps is to regulate the concentration of ions in intracellular compartments as, for example, in the case of the ubiquitous H^+^ ATPase responsible for the acidification of endosomes and other organelles [67, 68].

Despite their importance in a number of fundamental biological processes, few studies have addressed the effects of manipulating the activity of ion pumps in neural development; even fewer were focused on mammalian corticogenesis. For example, the Na^+^/K^+^ and Ca^2+^ ATPases have been shown to mediate dendritic outgrowth of mammalian cortical neurons [69] and midline signaling in zebrafish embryos [70]. Loss of function of the H^+^ ATPase in *Xenopus *has been shown to inhibit development and regeneration [71, 72], while gain of function had the opposite effect [71].

Due to their multiple roles [67, 68], it is currently difficult to determine to which extent the effects induced by manipulations of ATPases are primarily due to a change in membrane potential as compared to other functions including endocytosis, trafficking, and signaling. Nevertheless, several observations suggest that the latter functions may be the most relevant ones during development. In particular, manipulations of the Na^+^/K^+^ ATPase during dendritogenesis of rat cortical neurons were not accompanied by a change in membrane potential but rather by a change in Ca^2+^/calmodulin-dependent protein kinase signaling and cAMP-responsive element gene expression [69]. Moreover, the inhibition of Ca^2+^ ATPases that was shown to induce developmental defects in zebrafish was achieved by manipulating pumps specifically of the endoplasmic reticulum leading to increased intracellular Ca^2+^ and, thus altering the complex Ca^2+^-dependent signaling events occurring during development more than changing *V*_mem_ proper [70]. Finally, with regard to the role of the H^+^ ATPase various laboratories have independently shown that its inhibition affects the transduction of important signaling molecules, such as Notch [73, 74] and Wnt [75], that are known to control proliferation, tissue patterning, and development throughout the animal kingdom [76–79].

While essentially all experiments on the role of the H^+^ ATPase in stem cell differentiation were performed in non-mammalian species, recent evidences from our laboratory could extend the role of this proton pump in Notch signaling during mammalian cortical development [80]. In these experiments, a dominant-negative subunit of the H^+^ ATPase was overexpressed during mouse embryonic development in NSC triggering their premature differentiation through a reduction of endogenous Notch signaling [80]. These and other experiments [73, 74, 80] support the notion that intracellular cleavage of activated Notch requires trafficking through acidic endosomes [81–83], but the intrinsic difficulties in distinguishing between cell autonomous versus extrinsic effects, and reports showing that Notch signaling may not require endocytosis [84–86], have led to a long debate in the field. Nevertheless, the fact that ATPases can have multiple effects at the level of (i) the cell biophysical state, (ii) signaling of differentiation molecules, and (iii) cell cycle length, places these enzymatic complexes in an ideal position to control the differentiation of NSC during mammalian corticogenesis. 

Cation-chloride cotransporters are a family of membrane proteins that use the Na^+^/K^+^ transmembrane electrochemical gradient to transport Cl^−^ against its gradient. This family is composed by seven members, most of which are expressed in neurons, with only one K^+^ (KCC4) and one Na^+^ (NKCC1) coupled cotransporters being detected in the proliferative zones of the developing cortex and being responsible for pumping Cl^−^ outside or inside the cell, respectively [49, 50, 87]. KCC4 expression is specific for the VZ and SVZ and its levels seem to increase during development from E12 to E14 and disappear perinatally [50]. NKCC1 expression in NSC similarly increases during development, but it then switches from NSC to neurons before birth [50].

The high expression of NKCC1 in the embryonic VZ [50] provides an explanation for the high [Cl^−^]_i_ underlying GABA_A_ depolarization response in AP [50, 88]. In addition to NSC, NKCC1 is also highly expressed in immature cortical neurons from E18 to the first postnatal week [49, 50] while KCC2 shows a marked increase only after the first postnatal week [45, 49, 50]. These changes in the composition of Cl^−^ transporters during the first weeks of life are probably the cause for the reduced [Cl^−^]_i_ responsible for the excitatory versus inhibitory effects of GABA [88–91].

## 3. Membrane Potential and Proliferation versus Differentiation of NSC

The primary role of the concerted action of ion channels and pumps is to regulate the *V*_mem_ of cells and, thus, it is reasonable to assume that their effect on proliferation of NSC should be interpreted in the context of this function.

Various groups have measured the membrane potential of mammalian NSC during embryonic development by different approaches resulting in *V*_mem_ values that ranged from a maximum of −40 mV to a minimum of −70 mV [21, 25, 26, 50, 88, 92–94]. Because a higher proliferative activity is known to correlate with a less negative, or depolarized, *V*_mem_ [1, 7, 9] and NSC, in particular BP, lengthen their cell cycle as development proceed [12, 95], it would be expected that the different *V*_mem_ measured by the different authors should reflect the use of NSC at different developmental stages. Reinforcing this expectation, more negative, or hyperpolarized, *V*_mem_ during development may also be deduced from the fact that (i) adult NSC tend to be more hyperpolarized than embryonic NSCs [22], (ii) the activity of the K^+^/Na^+^/2 Cl^−^ transporter decreases during development [88], and (iii) developmentally regulated growth factors and signaling molecules influencing the cell cycle also influence the activity of ion channels [32, 96]. Yet, while comparing the measurements performed at different developmental stages, or from different regions of the central nervous system that contain, or lack, BP, [21, 25, 26, 50, 88, 92–94] we were unable to detect any specific trend. 

Certainly, the lack of evidence for a change in *V*_mem_ during development, and in particular between AP and BP, should not be considered as an evidence for its lack since this comparison has never been directly pursued. Many of the reports discussed above are consistent with the view that an artificial depolarization of NSC may increase their proliferative potential and delay neurogenesis, but some are not and reconciling them is particularly difficult if one considers the diverse approaches and experimental condition used among studies including NSC of different origins and culture conditions, diverse pharmacological approaches to manipulate the activity of various ion channels or pumps without necessarily measuring an effect on *V*_mem_ or, even less so, cell cycle length. Moreover, in nearly all studies discussed, it is difficult to assess whether hyperpolarization is a cell-intrinsic feature of a given subpopulation of differentiating NSC, such as BP that increase in number during development, or, alternatively, an overall characteristic of tissues at different embryonic stages, as would be expected from the fact that ion concentrations in the cerebrospinal fluid fluctuate during mammalian development [97]. In fact, discriminating between these possibilities would require the measurement and manipulation of *V*_mem_ concomitantly in two coexisting subpopulations of cells, such as multipotent AP and more committed BP, at one given developmental time. Clearly, the ideal conditions to performing such experiments are those in which other bioelectric features of NSC, including capacitance, conductivity, and electric coupling mediated by junctions are preserved within an intact tissue. 

The technical limitations intrinsic in these experiments are daunting, but recent developments provide the key towards addressing the role of *V*_mem_ in mammalian neurogenesis.

## 4. Conclusions

For many years the lineage of NSC during mammalian corticogenesis has been indirectly inferred from fixed tissues or retrospectively deduced upon S-phase labeling in vivo. Only recently has the establishment of time-lapse videomicroscopy and transgenesis evolved to the point that direct visualization of AP and BP divisions in organotypic slice cultures became possible [38–40]. Moreover, the identification of molecular markers for BP [37], the generation of transgenic reporter mice allowing their visualization in alive tissues [98, 99], and new methods to genetically manipulate individual cells in brain cortical slices [100] while also monitoring G1/S/G2 progression [101] currently allow us to directly investigate the role of ion channels, pumps, and their effects on membrane potential during mammalian corticogenesis at the single-cell level. Overcoming the use of dissociated cells cultures and uncertainties with regard to the identity of different progenitors subtypes, these powerful new tools may allow us to reveal a new role of bioelectric signaling in NSC differentiation and likely reconcile the different reports that were discussed in this paper.

Similarly, great emphasis on the role of apicobasal polarity in AP/BP transition and neurogenesis has recently come to light in particular in the context of asymmetric cleavage plane orientation [102], subcellular localization of cell cycle regulators [103], and evolution of the mammalian brain [104]. This in turn triggers the question as to whether or not the subcellular localization of certain channels or pumps, rather than their absolute expression levels, might be important for cell fate change. Unfortunately, however, identification of ion pumps and channels in tissues has been historically established by electrophysiology or, alternatively, by in situ hybridization, neither of which provides any information about protein localization. When immunohistochemical characterization was undertaken [23, 25, 44, 54, 55, 64, 65], this was performed either on dissociated cells where apicobasal polarity is lost or in intact tissue but exclusively for the Na-K-Cl cotransporter that showed no preferential localization in the apicobasal axis [50].

Apparently, the two big fields comprising (i) cell biologists studying the cell cycle, lineage, and polarity of NSC and (ii) physiologists studying their channels, pumps, and membrane potential have seldom met. We hope that our paper may underline the importance of this interdisciplinary field.
